# Influence of Light and Water Activity on Growth and Mycotoxin Formation of Selected Isolates of *Aspergillus flavus* and *Aspergillus parasiticus*

**DOI:** 10.3390/microorganisms8122000

**Published:** 2020-12-15

**Authors:** Eva-Maria Priesterjahn, Rolf Geisen, Markus Schmidt-Heydt

**Affiliations:** Max Rubner-Institut Karlsruhe Federal Research Institute for Nutrition and Food, Haid-und-Neu-Str. 09, 76131 Karlsruhe, Germany; Eva-Maria.Priesterjahn@mri.bund.de (E.-M.P.); rolf.geisen@mri.bund.de (R.G.)

**Keywords:** *Aspergillus*, aflatoxin, cyclopiazonic acid, light, water activity

## Abstract

*Aspergillus flavus* and *A. parasiticus* are the main causes of aflatoxin contamination in various foods, particularly grains, as they can thrive in environments with lower water activity and higher temperatures. The growth of *Aspergillus* and the formation of the mycotoxins aflatoxin and cyclopiazonic acid are strongly influenced by environmental stimuli and can be reduced by modulating parameters such as water activity, pH, temperature and light during the storage. This study has two objectives—on the one hand, to assess how global warming and an increase in exposure to sunlight affect growth and mycotoxin formation, and on the other hand, how the findings from these experiments can be used to reduce fungal growth and mycotoxin formation in stored foods. Using growth substrates with two different water activities (a_w_ 0.95, a_w_ 0.98), together with a light incubation device consisting of different chambers equipped with diodes emitting visible light of five different wavelengths (455 nm, 470 nm, 530 nm, 590 nm, 627 nm) plus white light, we analyzed the growth and mycotoxin formation of selected *Aspergillus flavus* and *A. parasiticus* isolates. It was shown that light with a wavelength of 455/470 nm alone, but especially in combination with a lower water activity of a_w_ 0.95, leads to a significant reduction in growth and mycotoxin formation, which was accompanied by reduced transcriptional activity of the responsible mycotoxin biosynthetic genes. Therefore, these results can be used to significantly reduce the growth and the mycotoxin formation of the analyzed fungi during storage and to estimate the trend of fungal infestation by *Aspergillus flavus* and *A. parasiticus* in water activity- and light exposure-equivalent climate change scenarios. Mycotoxin-producing aspergilli can be effective and sustainably inhibited using a combination of short-wave light and lowered water activity in the substrate. A higher annual mean temperature accompanying climate change may lead to an increased spread of aflatoxin-producing fungi in areas that were previously too cold for them. On the other hand, there will be regions in the world where contamination with aflatoxin-producing fungi will be reduced due to increased drought and sun exposure.

## 1. Introduction

Mycotoxins, which are produced by different species of filamentous fungi, are common worldwide. Contaminated foods should be rejected for reasons of food safety, at the expense of the food supply of a steadily growing world population. The global food loss due to contamination with mycotoxins is estimated by the Food and Agriculture Organization (FAO) to be more than 25%, a value that apparently greatly underestimates the real situation [1]. In particular, the aflatoxins produced by some *Aspergillus* species should be emphasized here as health-relevant mycotoxins. Particular aflatoxin producers that cause problems in food are the species *Aspergillus flavus*, which mainly produces the B-aflatoxins B_1_ and B_2_, as well as *A. parasiticus*, which can also produce the G-aflatoxins G_1_ and G_2_ [2,3,4]_._ The former occurs mainly in cereals such as corn and wheat [5], the latter in cotton, Brazil nuts, peanuts and soil [6]. Both species are found particularly in regions with a warm climate such as Sub-Saharan Africa, Iran, Iraq, India and Southeast-Asia [7], but in more recent times they have also been found in Southern Europe due to global warming [8]. Aflatoxin B_1_ has been classified as carcinogenic by the FAO (IARC, 2012) [9,10,11]. After ingestion, aflatoxin is metabolized to the highly reactive aflatoxin B_1_ 8,9-epoxide, which forms DNA adducts. In addition, in mammals aflatoxin B_1_ is hydrolyzed to another toxic compound called aflatoxin M_1_, which is excreted with milk, in a process called carry-over [12]. Both the uptake of the parent mycotoxin and exposure to the excretion product M_1_ via milk can trigger reduced growth rates in children, liver carcinoma [13] and the disturbance of the immune system [14,15]. In regions affected by aflatoxin-producing fungi, the aflatoxin levels can often be high and in several cases greatly exceed the legal limits [16]. Another toxic secondary metabolite produced by various genera of fungi including *Aspergillus* is the indole-tetramic-acid, cyclopiazonic acid (CPA) [17,18,19,20]. CPA is a potent inhibitor of the Ca^2+^ ATPase in the sarcoplasmic reticulum, has neurotoxic effects and leads to liver damage and degenerative changes in the kidneys [21,22]. It should be noted that not only aflatoxigenic *Aspergillus* species produce CPA—it is also produced by atoxigenic isolates such as those used as biocontrol organisms to compete with aflatoxigenic strains [20].

Mycotoxin contamination is not limited to post-harvest problems but can occur at several steps in the food processing chain, such as in the field due to infestation of the plants by aflatoxigenic fungi and during the subsequent storage under causal conditions or due to carry-over. Various environmental parameters influence whether a host plant is infected by a fungus and how much mycotoxin is produced by the fungus on the plant. These parameters include, for example, the susceptibility of the host plant, the composition and quality of the soil, the temperature and water availability (water activity/vapor pressure of water), harmful insects, competitors for fungi and the duration and intensity of solar radiation [23]. Aflatoxins consist of a substituted coumarin ring, which, depending on the aflatoxin, condenses on either a pentanone ring or a lactone ring. Aflatoxin biosynthesis is a multi-step process in which the polyketides are synthesized from acetate and malonate subunits [24]. The genes coding for mycotoxins such as aflatoxins are usually organized in the form of gene clusters. The transcription activity of such clusters is regulated by central regulatory genes, which are activated by specific transcription factors and influenced by physiological conditions [25,26,27,28]. Well-known physiological conditions that control aflatoxin biosynthesis are temperature, pH value, nitrogen and carbon sources and light [29,30,31,32], which are perceived via specific signaling pathways [33].

The influence of light is perceived, for example, by the blue light receptor signal system the “White-Collar-Complex”, first described by Crosthwaite and coworkers in *Neurospora crassa* [34]. Other research groups later described the influence of light on growth and mycotoxin formation in various species of *Penicillium*, *Aspergillus*, *Alternaria* and *Fusarium* [35,36,37,38,39,40]. It is known that in *A. nidulans* blue- and red-light receptor signaling is connected to the HOG (high osmolarity glycerol) MAP kinase pathway [41]. This pathway, which is regularly induced at lower water activities in response to drought stress, was also analyzed in species of *Penicillium* [42,43] and *Alternaria* [44]. In addition, in other works the connection between the signaling pathway for osmotic stress and light perception has also been examined for the plant pathogens *A. alternata* and *Botrytis cinerea* [37,45].

The increasing spread of aflatoxin-producing fungi due to climate change and the connection between these two important signaling pathways in fungi, HOG and light regulation, were the reason for the experimental setup in this study. In this setup, the combined effects of selected water activities and light wavelengths on the formation of the mycotoxins aflatoxin and cyclopiazonic acid were analyzed using three common isolates of *A. flavus* and *A. parasiticus*. The results obtained from the experiments are intended to provide an assessment of how global warming and an increase in light radiation affect growth and mycotoxin formation. We also aim to assess how the findings of the experiments can be used to reduce fungal growth and mycotoxin formation, especially during food storage.

## 2. Materials and Methods

### 2.1. Fungal Strains and Growth Conditions

For the growth experiments one non-aflatoxin producing *A. flavus* isolate and one aflatoxin B_1_/B_2_ producing *A. flavus* isolate was used, both producing cyclopiazonic acid, and one *A. parasiticus* isolate producing the aflatoxins B_1_/B_2_ and G_1_/G_2_. These representative isolates were chosen for their constant growth and mycotoxin formation over a period of time under standard laboratory conditions. Our analyses were focused on *pks*A (a key gene for aflatoxin production) and *cpa*A (a key gene for cyclopiazonic acid production). We carried out gene expression analyses by means of real-time PCR, determination of the growth rate by diameter measurement and mycotoxin quantification by HPLC. The two aflatoxin-producing strains *A. flavus* MRI19 and *A. parasiticus* BFE84 belonged to the culture collection of the Max Rubner-Institut, whereas *A. flavus* ATCC96045 was purchased from ATCC (ATCC: The Global Bioresource Center, atcc.org) as a non-aflatoxin producing strain. This strain, however, is able to produce CPA. The strains were grown in triplicate on potato dextrose agar (PDA) plates (25 g/L potato—dextrose agar, 10 g/L agar) modified with two different amounts of glycerol in order to adjust the water activity to 0.95 and 0.98 a_w_, as described in [46]. PDA was used because in our preliminary experiments the selected fungal isolates reliably showed high mycotoxin formation and growth on this substrate compared to other good growth substrates such as yeast extract sucrose (YES) or malt extract glucose agar (MGA). Regardless of the extent of growth and toxin formation, the experimental outcome was comparable on all growth media (not shown). Spore suspensions containing 1 × 10^5^ spores/mL were used for single-point inoculation in the center of the agar plates. Inoculations were carried out in biological triplicates. The cultures were incubated up to 10 days at different light wavelengths at room temperature (+/− 25 °C). The light box, which has been described before [47], was equipped with six incubation chambers containing diodes which emit wavelengths from red (627 nm, 7700 lx); white (17,750 lx); yellow (590 nm, 6400 lx) and green (530 nm, 7250 lx) to blue (470 nm, 2357 lx) and royal blue light (455 nm, 3350 lx). Daily growth and morphology of the colonies were analyzed by measuring the diameters twice, perpendicular to each other. Standard deviation between biological triplicates was calculated.

### 2.2. Isolation of RNA, cDNA Synthesis and Real-Time PCR

Mycelium samples were prepared for RNA isolation using the RNAeasy Plant Mini kit (Qiagen, Hilden, Germany). A volume of 6.5 µL β-mercaptoethanol and 650 µL RLC buffer solution was added to 80 mg of the mycelium and were homogenized for 2 × 30 s at 5 m/s using a MP FastPrep-24 Tissue and Cell Homogenizer (MP Biomedicals Germany GmbH, Eschwege, Germany). The samples were mixed thoroughly and incubated for 3 min at 56 °C. All further procedures were carried out according to the manufacturer’s instructions for the kit (RNAeasy Plant Mini kit (Qiagen, Hilden, Germany). cDNA synthesis was performed using 12 µL of RNA sample along with the Omniscript Reverse Transcription kit (Qiagen, Hilden, Germany) according to the manufacturer’s instructions. Gene expression analysis was performed with iQ™ SYBR^®^ Green Supermix using a Bio-Rad iQ™5 Multicolor Real-Time PCR Detection System (Bio-Rad Laboratories GmbH, Hercules, CA, USA). Primer pairs resulting in amplificate sizes of about 1000 bp (external) and about 100 bp (internal) were designed and then purchased at biomers.net GmbH (Ulm, Germany). The primer set had the following nucleotide sequences: pksA_extern_for (5′-cacaatggcgccctcttcacgc-3′), pksA_extern_rev (3′-gttagttcctgccaccgccg-5′); pksA_intern_for (5′-gaccaccgaaagcttttgggac-3′), pksA_intern_rev (3′-cacatgcgtgttgatgtcccac-5′), cpaA_extern_for (5′-cctgcgagcactactttatcgg-3′), cpaA_extern_rev (3′-ccatactcctccaaagttatggg-5′), cpaA_intern_for (5′-ggtgaaatcacctaggagtggc-3′), cpaA_intern_rev (3′-ggtccagcctccagaaggc-5′). After an activation step of 10 min at 95 °C, real-time PCR was performed using the following temperature regime: 95 °C for 20 s, 55 °C for 40 s and 72 °C for 60 s for 40 cycles. Standard curves for all analyzed fragments were generated using the external primer pairs for the respective genes of interest. The concentration of the PCR products for the standard curve was determined using a Qubit 3 Fluorometer using the dsDNA HS Assay Kit (Thermo Fisher Scientific Inc., Waltham, MA, USA). The copy numbers were calculated and stock solutions were diluted between 10^2^ to 10^10^ copy numbers/µL. Aliquots of the dilution series were used during all real-time PCR setups. The concentrations of the samples were calculated using Bio-Rad iQ5 Optical system software, calculating and including the generated standard curve automatically. All samples had three technical and six biological replicates, which were then pooled.

### 2.3. Quantification of Aflatoxins and Cyclopiazonic Acid Using High-Performance Liquid Chromatography (HPLC)

For determination of aflatoxin B_1_, B_2_, G_1_ and G_2_ and CPA biosynthesis, three agar plugs (diameter 1 cm) of three colonies were taken in triplicates from the region between center and the edge of the colony with the aid of a sterile corer. Agar plugs were transferred into 2-mL micro-reaction tubes and 750 µL of chloroform were added. Mycotoxins were extracted for 30 min at room temperature on a rotary shaker; the agar plugs were discarded and the chloroform extract was evaporated to dryness in a vacuum concentrator (Speed Vac, Savant Instruments, Farmingdale, NY, USA). Residues were subjected to HPLC determination. Quantitative determination of aflatoxins and CPA of each of the three biological replicates using HPLC was performed on a Hitachi Chromaster HPLC system (VWR International GmbH, Darmstadt, Germany) equipped with an auto-injector 5260, column oven 5310, pump 5160, DAD 5430 and fluorescence detector 5440. For aflatoxin detection the column oven was set to 40 °C; the fluorescence detector was set to an excitation of 365 nm and an emission of 450 nm. The flow rate was 0.7 mL/min and the injection volume was 10 μL. Solvent A consisted of 250 mM acetonitrile, solvent B of water and solvent C of methanol. Separation was carried out on a Hitachi LaChrom C18 (150/4.6–3 µm) reversed-phase column (VWR International GmbH, Darmstadt, Germany) using the following gradient (*v*/*v*): 0 min—20 min shift from solvent A 10% and solvent B 60% to solvent C 30%. For CPA detection, the column oven was set to 25 °C; the fluorescence detector was set to an excitation of 333 nm and an emission of 460 nm. The flow rate was at 0.8 mL/min and the injection volume was 10 μL. Solvent A consisted of 250 mM acetonitrile, solvent B of 0.1% formic acid. Separation was carried out on a Kinetex 2.6 µm Evo C18 100A (100 × 4.6 mm) column (Phenomenex, Torrance, CA, USA) using the following gradient (*v*/*v*): 0 min—solvent A 5%, solvent B 95%, 5 min—solvent A 50%, solvent B 50%, 15 min—solvent A 95%, solvent B 5%, 17 min—solvent A 5%, solvent B 95%, 22 min—solvent A 5%, solvent B 95%. All standards used were obtained from Sigma (Taufkirchen, Munich, Germany) with a purity of ≥98%. Data collection and handling was done with EZChrome Elite 3.2.

### 2.4. Statistical Calculation

The statistical analysis of growth values in relation to the control (growth without treatment) was carried out using the hypothesis test. The *p*-value for the growth rates was calculated with a significance level of α = 0.05. Additionally, to measure the dispersion of biological replicate samples relatively to its mean, standard deviations of means were calculated. For mycotoxin production and gene expression analysis, the paired *t*-test was used to calculate the average between treated samples and the control. In cases where measured mycotoxin concentrations in the samples were too low, no statistical significance tests were performed.

## 3. Results and Discussion

### 3.1. Growth of Aspergillus in Relation to Water Activity and Light

**Figure 1:**
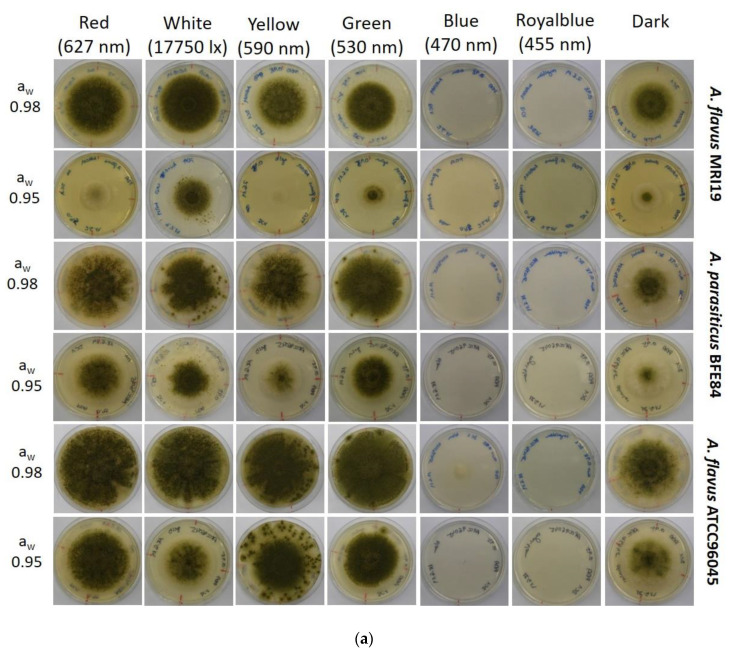

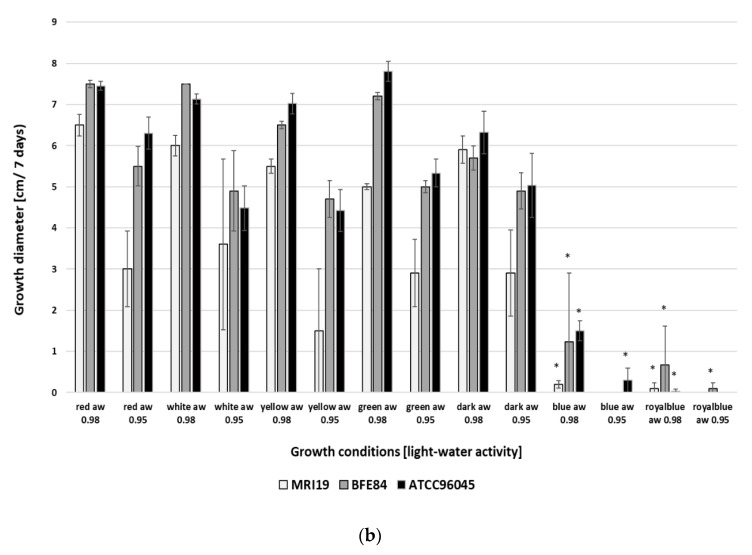
(**a**) Phenotypic growth of the analyzed *Aspergillus* isolates dependent on different combinations of light and water activity. (**b**) Growth rates of the analyzed *Aspergillus* isolates dependent on different combinations of light and water activity. Standard deviations are given for each parameter. * = The growth was significantly reduced in relation to the control (dark).

The influence of combinations of two different water activities (a_w_) and light with five different wavelengths plus white light on the growth of *A. flavus* MRI19, *A. flavus* ATCC96045 and *A. parasiticus* BFE84 was analyzed. These two environmental influencing factors are of particular importance with regard to global warming and the natural habitat of aflatoxigenic fungi, which is even now often exposed to light and drought stress. The strains were inoculated on PDA adjusted to a_w_ 0.98 and a_w_ 0.95 with glycerol and incubated at 25 °C under the influence of different wavelengths of light. Figure 1a,b shows the phenotypic and quantitative growth differences of the three *Aspergillus* isolates under different combinations of light and water activity. The growth of the three aspergilli analyzed was generally reduced, with lower water activity in the growth media. This is in accordance with the findings of Gibson et al. [48], who showed that *Aspergillus* grows particularly well at moderate temperatures and high water activity conditions. Experiments with other fungal species, mainly from the genus *Penicillium*, showed that both growth and mycotoxin production on substrates without adaption of the water activity were strongly influenced by the influence of different light wavelengths alone [36,38,47]. Sporulation and the formation of mycelia were also significantly affected in this context. The combination of slight drought stress, which is mimicked by the lower water activity of the substrate, together with light radiation, however, shows a clear promotion of the inhibition of growth for the analyzed *Aspergillus* species. In particular, a water activity of a_w_ 0.95 in the growth substrate, together with short-wave light radiation (455/470 nm), led to a significant reduction or even a complete cessation of fungal growth. However, some of the spores showed an increased tolerance to drought stress and short-wave light radiation because they survived the procedure, and this led to tiny colonies after prolonged incubation (data not shown). These adapted spores which showed a higher resilience were positively selected by the harmful growth conditions particularly in case of the species *A. parasiticus*. *A. parasiticus* BFE84 and the non-aflatoxin producing strain *A. flavus* ATCC96045 showed higher growth rates under the given conditions than the aflatoxin-producing *A. flavus* strain MRI19. In general, longer wavelengths (530/627 nm) appear to support the growth of the fungi, whereas incubation under short wavelengths (455/470 nm) reduced growth persistently over an observation period of at least two weeks.

### 3.2. Aflatoxin Biosynthesis of Aspergillus in Relation to Water Activity and Light

**Figure 2:**
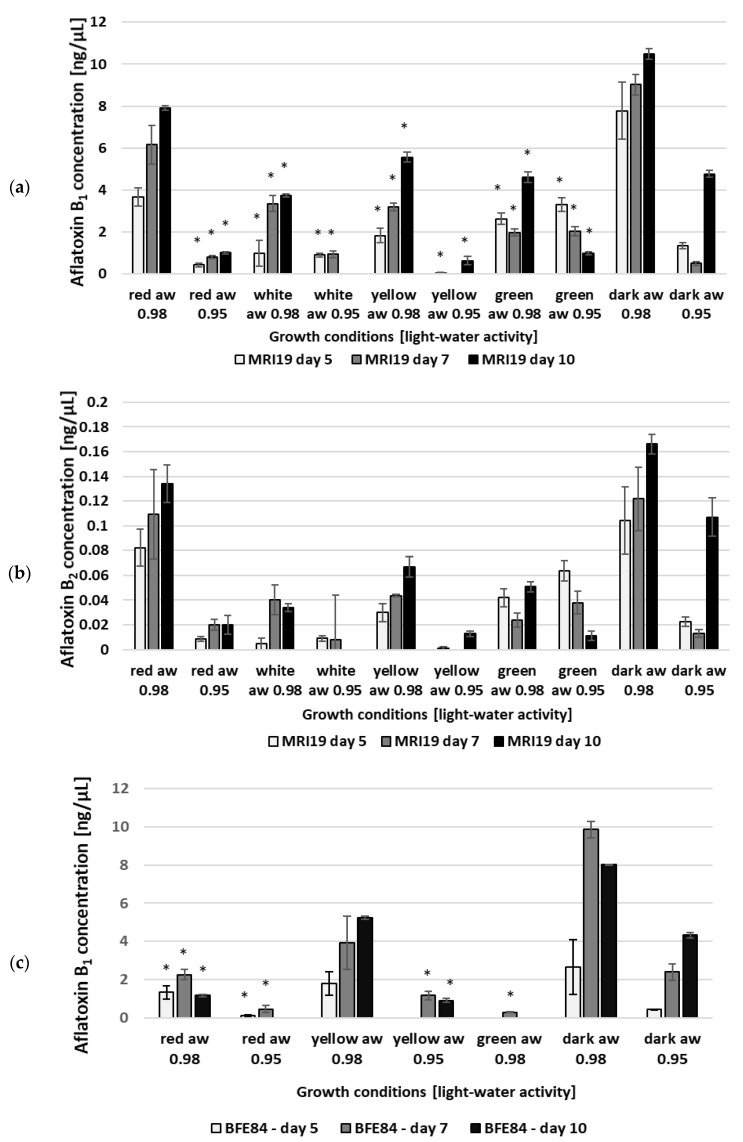

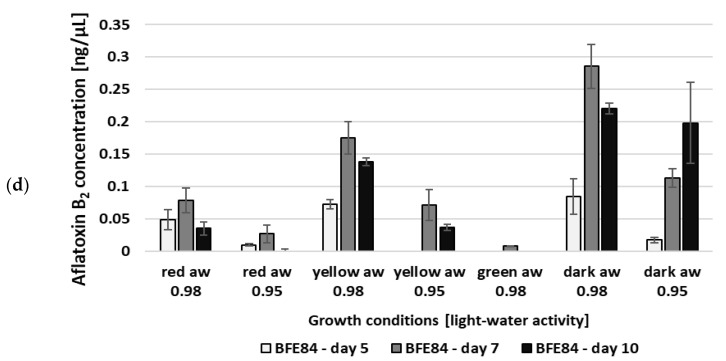
Aflatoxin B_1_ (**a**) and B_2_ (**b**) formation by *Aspergillus flavus* MRI19; aflatoxin B_1_ (**c**) and B_2_ (**d**) formation by *Aspergillus parasiticus* BFE84, dependent on different combinations of light and water activity. No aflatoxin was detectable in the case of *A. parasiticus* BFE84 under white light, a_w_ 0.98 and a_w_ 0.95; green light and a_w_ 0.95; and blue/royal blue light. Standard deviations are given for each parameter. * = The toxin production was significantly reduced in relation to the control (dark). For B_2_-aflatoxins the measured values were too low for statistical significance analysis.

**Figure 3:**
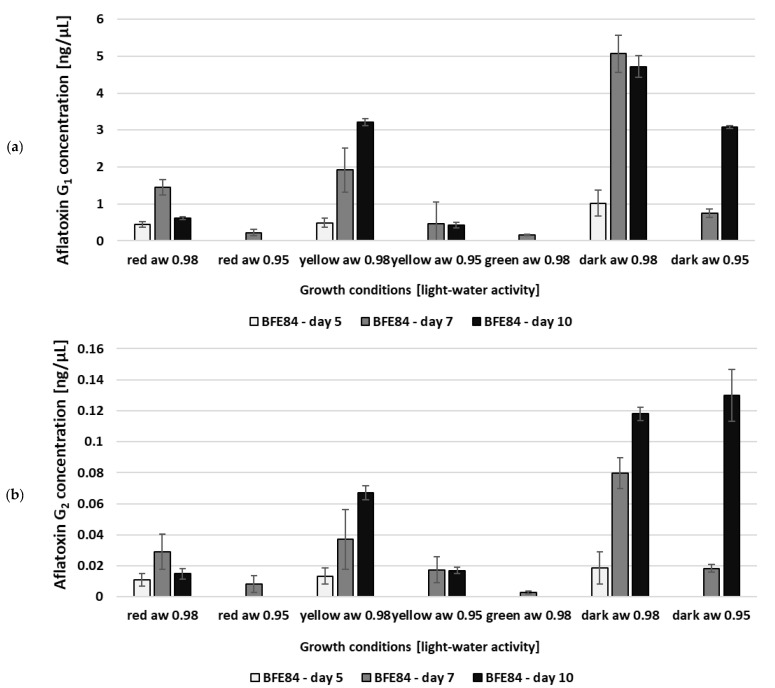
Aflatoxin G_1_ (**a**) and G_2_ (**b**) formation by *Aspergillus parasiticus* BFE84, dependent on different combinations of light and water activity. No aflatoxin formation was detectable under white light, a_w_ 0.98 and a_w_ 0.95; green light and a_w_ 0.95; and blue/royal blue light. Standard deviations are given for each parameter. The measured values were too low for statistical significance analysis.

The combination of moderate drought stress and different light wavelengths also showed a substantial effect on mycotoxin production, as shown in Figure 2 and Figure 3. Under the influence of only one parameter—light or water activity—mycotoxin formation was less affected. The combined effect of both influencing factors—short-wave light (455/470 nm) together with lower water activity (a_w_ 0.95)—however, leads to a significant reduction in growth and a complete inhibition of mycotoxin formation. The aflatoxin B_1_/B_2_ formation of the *A. flavus* MRI19 and *A. parasiticus* BFE84 isolates over a period of ten days is shown in Figure 2a–d and the formation of aflatoxin G_1_/G_2_ by *A. parasiticus* is shown in Figure 3a,b. The highest aflatoxin concentrations could be measured in the growth of *Aspergillus* without light radiation. This leads to the assumption that aflatoxin formation in the dark is preferred and that aflatoxin obviously does not help to compensate for the harmful effects of light radiation, as described in the case of the mycotoxin citrinin produced by *P. verrucosum* [49,50]. Incubation under long-wave light (627 nm, red light) leads to a good reduction in aflatoxin formation of about 20% compared to dark conditions. However, the sporulation, mycelial phenotypical habitus and mycotoxin formation were still most similar to the samples incubated in the dark, followed by samples incubated at medium wavelengths. Under white, yellow and green light (depending on the fungal strain) the mycotoxin concentration was reduced even further. This observation differs from the results of Moussa et al. [40], who described an induction of aflatoxin B_1_ formation in *A. flavus* irradiated with white light. The reason for this discrepancy could be that they did not use LED light, which is more monochromatic “cold-white” light compared to Edison lightbulbs, which emit more yellow “warm white” light. Other groups found that white light harms the formation of sclerotia in *A. flavus* growing in liquid medium and to some extent the production of secondary metabolites [51,52]. It is already known that the reaction of the fungus to light depends on physical parameters associated with light such as the wavelength, the intensity, the duration of the radiation and thus the energy to which the fungus is exposed [38,47]. In addition, fungi that are well adapted to highly UV-exposed habitats such as melanin- or carotenoid-pigmented fungi that occur in crop fields in parallel with *Aspergillus* may be less sensitive to light radiation.

### 3.3. Cyclopiazonic Acid (CPA) Biosynthesis of Aspergillus in Relation to Water Activity and Light

**Figure 4:**
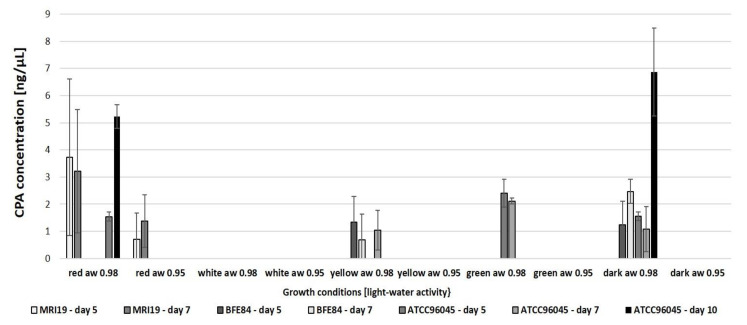
Cyclopiazonic Acid (CPA) formation in the analyzed *Aspergillus* isolates, dependent on different combinations of light and water activity. No CPA was detectable on day 10 by MRI19 and BFE84 under white light at a_w_ 0.98, a_w_ 0.95 and green light at a_w_ 0.95 and blue/royal blue light. Standard deviations are given for each parameter. The measured values were too low for statistical significance analysis.

In addition to the formation of aflatoxins, we were interested in the formation of cyclopiazonic acid (CPA) by the analyzed aspergilli. *A. flavus* strain ATCC96045 does not produce aflatoxins, although it does produce CPA (Figure 4). Similarly to the formation of aflatoxins, CPA production by the fungal strains was possible after irradiation with red and yellow light and in the dark. However, there were major differences between the strains analyzed. Although the CPA formation in the two aflatoxin-producing strains generally remained low, the concentration in the ATCC96045 strain increased, particularly at the end of the experiment.

### 3.4. Transcriptional Activity of Mycotoxin Biosynthetic Genes in Aspergillus in Relation to Water Activity and Light

**Figure 5:**
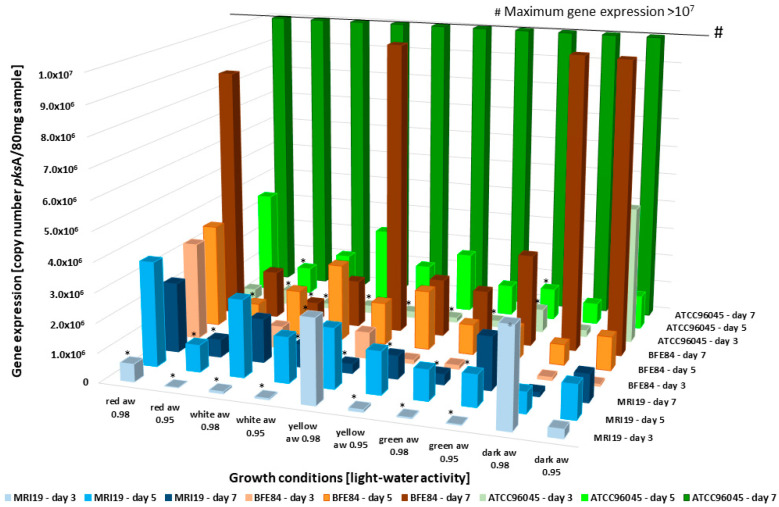
Expression of the *pks*A gene over 3 days in the analyzed *Aspergillus* isolates, dependent on different combinations of light and water activity. * = The gene expression was significantly reduced in relation to the control (dark).

In general, it must be considered that toxin formation is an accumulated value if toxins are not metabolized further or are degraded by the fungus, whereas gene expression is a “snapshot” and thus depends on mRNA stability and other factors that are continuously changing [53,54]. Depending on the physiological status of the fungal cell, the gene expression shows a direct correlation to mycotoxin formation or has more of a predictive value [32]. Nevertheless, it has been shown that gene expression can be used as an early indication of active mycotoxin biosynthesis [55]. In order to investigate the phenotypic mycotoxin formation in relation to the transcriptional activity of aflatoxin biosynthetic genes, real-time PCR gene expression analyses were carried out, which targeted the polyketide synthase gene *pks*A from the aflatoxin cluster and the hybrid PKS-NRPS gene *cpa*A, which is involved in cyclopiazonic acid formation. The gene expression analyses were compared to the measured aflatoxin data, and as shown in Figure 5, the *pks*A expression was reduced even under slight drought stress. The highest *pks*A expressions were measured after red and yellow light irradiation, as well as in the dark and with a water activity of a_w_ 0.98. In these cases, the expression of *pks*A was already increased on day 3, whereas the upregulation under the other conditions began within the fifth day. The transcriptional activity of the *pks*A gene increased proportionally to the formation of aflatoxin under the given conditions and with the duration of the experiment. A lower water activity of the medium was associated with reduced toxin formation and reduced expression of the polyketide synthase gene *pks*A. In addition, the differences in gene expression and aflatoxin formation with regard to light wavelength are interesting. Toxin formation and *pks*A gene expression were paralleled under the influence of red light and yellow light radiation and samples incubated in the dark. *Pks*A expression was activated immediately under these conditions, which led to earlier aflatoxin production. In general, the data show that toxin formation is negatively affected by short-wave light. It has already been described earlier that genes of different secondary metabolites are often mutually regulated in different light conditions. In this context, some secondary metabolites are induced and others are reduced [56,57].

Jayashree et al. observed that aflatoxin is formed in particular under conditions that cause oxidative stress for the fungus [58]. Oxidative stress can be induced by chemicals such as hydrogen peroxide [59], but also by short-wave light radiation [60]. A connection between oxidative stress and aflatoxin formation as a kind of compensation mechanism would be assumed, as shown in the case of the mycotoxin citrinin produced by the species *Penicillium verrucosum* [49,50]. In this species, citrinin acts as a radical scavenger, protecting the fungus from oxidative stress induced by short-wave light radiation. For aflatoxin, the physiological benefit for the producing fungus is not yet so clear. Kolliputi et al. found that oxygen radicals are required for aflatoxin formation, so its formation can be induced by oxidative stress [61]. For this reason, it was expected that the formation of aflatoxin would be activated by the short-wave radiation of blue light, but this was not the case in the experiments. It is possible that the oxidative stress induced by the applied short-wave light was below the threshold for an induction of aflatoxin biosynthesis. Another interesting observation was that the non-aflatoxin producing *A. flavus* strain ATCC96045 exhibited remarkably high *pks*A expression at the 7th day, especially under red light, yellow light and dark growth conditions, i.e., the growth-promoting conditions. The *pks*A gene expression rates were more than two orders of magnitude higher compared to the two other aflatoxin producing strains, regardless of the fact that this *Aspergillus* strain is not able to form aflatoxin. The reason for its inability to produce aflatoxin is apparently due to deletions which were identified in various genes of the aflatoxin cluster during detailed sequencing analyses of the genome sequence of A. *flavus* ATCC96045 (data not shown).

**Figure 6:**
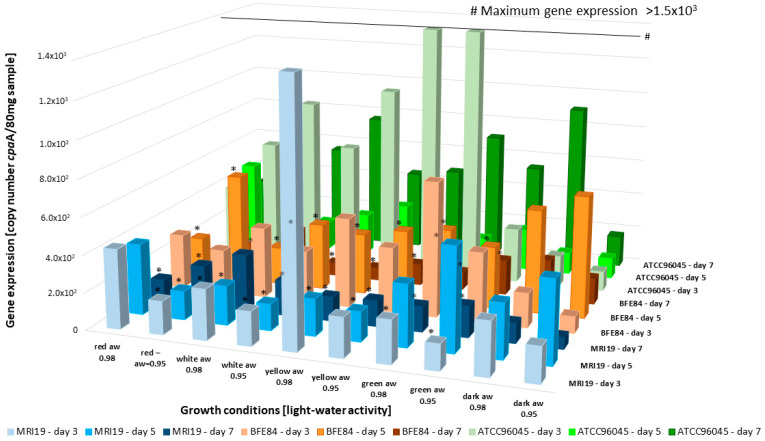
Expression of the *cpa*A PKS-NRPS gene over 3 days in the analyzed *Aspergillus* isolates, dependent on different combinations of light and water activity. * = The gene expression was significantly reduced in relation to the control (dark).

The gene expression analysis with the key gene for CPA formation, *cpa*A, a hybrid polyketide synthase/non-ribosomal peptide synthase gene [62], showed increased transcription activity in the strain *A. flavus* ATCC96045 (Figure 6) from the beginning. It is noteworthy that the gene expression under yellow light radiation and a_w_ 0.95 was high, whereas no CPA could be detected by HPLC. This suggests that some post-translational mechanisms could hinder CPA formation in a certain step of the biosynthesis, which again shows that the regulation of mycotoxin formation is a very complex process [53]. 

## 4. Conclusions

It is shown here that the formation of aflatoxin and cyclopiazonic acid by *Aspergillus flavus* and *Aspergillus parasiticus* can be greatly reduced by using a combination of lower water activity (a_w_ 0.95) and short-wave light (455/470 nm) and that this was paralleled by a reduced transcription activity of the corresponding mycotoxin biosynthetic genes. These conditions also led to the severe growth inhibition of all aspergilli analyzed. It is clear that these conditions can be controlled in particular in storage houses, rather than in the field. However, the results of the experiments reported in this study may allow for the prediction of how such conditions (increased sun exposure and drought) support the further spread of aflatoxin-producing fungi and the conditions that lead to increased or decreased aflatoxin production.

Global warming and cooling change at regular intervals depending on many variables, including solar activity, the amount of greenhouse gases such as CO_2_ in the atmosphere and indirect climate variables such as air exchange, humidity, evaporation capacity of surface water, etc. Considering the experimentally obtained data collected here, even if there were only a few isolates of *A. flavus* and *A. parasiticus*, it can be assumed, together with the optima for growth and aflatoxin biosynthesis reported in other publications [31,32,63], that an increase in drought and short-wave radiation (UV, blue) in areas that warm up and are heavily exposed to the sun leads to lower aflatoxin contamination. Overall, however, there will be further spread of aflatoxin-forming fungal species in areas that were previously too cold and dry and that are now becoming warmer and more humid due to global warming, as is already the case in parts of Europe.

Future analyses should further investigate the benefit of the formation of the secondary metabolites aflatoxin and CPA for *Aspergillus* and their possible involvement in physiological aspects such as virulence and adaption to habitat. This would enable the development of more effective and sustainable antifungal strategies.
